# RNA-based analysis of ALK fusions in non-small cell lung cancer cases showing IHC/FISH discordance

**DOI:** 10.1186/s12885-018-5070-6

**Published:** 2018-11-22

**Authors:** Claudia Vollbrecht, Dido Lenze, Michael Hummel, Annika Lehmann, Markus Moebs, Nikolaj Frost, Philipp Jurmeister, Leonille Schweizer, Udo Kellner, Manfred Dietel, Maximilian von Laffert

**Affiliations:** 1 Charité-Universitätsmedizin Berlin corporate member of Freie Universität Berlin, Humboldt-Universität zu Berlin, and Berlin Institute of Health, Institute of Pathology, Charitéplatz 1, 10117 Berlin, Germany; 2 German Cancer Consortium (DKTK), partner site Berlin, Germany; 3 Geman Cancer Research Center (DKFZ), Im Neuenheimer Feld 280, 69120 Heidelberg, Germany; 4 Medical Department, Division of Infectiology and Pneumology, Charité-Universitätsmedizin Berlin corporate member of Freie Universität Berlin, Humboldt-Universität zu Berlin, and Berlin Institute of Health, Augustenburger Platz 1, 13353 Berlin, Germany; 5 Charité-Universitätsmedizin Berlin corporate member of Freie Universität Berlin, Humboldt-Universität zu Berlin, and Berlin Institute of Health, Charité Comprehensive Cancer Center, Virchowweg 23, 10117 Berlin, Germany; 6 Johannes Wesling Klinikum Minden, Institute for Pathology, Hans-Nolte-Straße 1, 32429 Minden, Germany; 7 Berlin Institute of Health (BIH), Anna-Louisa-Karsch-Straße 2, 10178 Berlin, Germany

**Keywords:** Non-small cell lung cancer (NSCLC), Anaplastic lymphoma kinase (ALK), Fluorescence in-situ hybridization (FISH), Immunohistochemistry (IHC), Massive parallel sequencing (MPS), NanoString

## Abstract

**Background:**

Rearrangements of the anaplastic lymphoma kinase (ALK) belong to the promising targets in the therapy of advanced non-small cell lung cancer (NSCLC) and are predominantly detected by immunohistochemistry (IHC) and/or fluorescence in-situ hybridization (FISH). However, both methods occasionally produce discordant results, especially in so-called borderline (BL) cases, showing ALK FISH-positive signals in 10–20% of the tumor nuclei around the cutoff (15%). This leads to a diagnostic and thus to a therapeutic dilemma.

**Methods:**

We selected 18 unequivocal (12 ALK IHC/FISH-negative; 6 ALK IHC/FISH-positive) and 15 equivocal samples with discordant results between FISH (Abbott, Vysis LSI ALK Dual Color) and IHC (Ventana, D5F3), including cases with FISH-BL results, for further RNA based-analysis**.** To detect ALK rearrangement at the transcriptional level, RNA was analyzed using a targeted multiplex-PCR panel followed by IonTorrent sequencing and by direct transcript counting using a digital probe-based assay (NanoString). Sensitivity of both methods was defined using RNA obtained from an ALK-positive cell line dilution series.

**Results:**

Cases with unequivocal IHC/FISH results showed concordant data with both RNA-based methods, whereas the three IHC-negative/FISH-positive samples were negative. The four IHC-negative/FISH-BL-negative cases, as well as the five IHC-negative/FISH-BL-positive samples showed negative results by massive parallel sequencing (MPS) and digital probe-based assay. The two IHC-positive/FISH-BL-positive cases were both positive on the RNA-level, whereas a tumor with questionable IHC and FISH-BL-positive status displayed no ALK fusion transcript.

**Conclusions:**

The comparison of methods for the confirmation of ALK rearrangements revealed that the detection of ALK protein by IHC and ALK fusion transcripts on transcriptional level by MPS and the probe-based assay leads to concordant results. Only a small proportion of clearly ALK FISH-positive cases are unable to express the ALK protein and ALK fusion transcript which might explain a non-responding to ALK inhibitors. Therefore, our findings led us to conclude that ALK testing should initially be based on IHC and/or RNA-based methods.

## Background

Alterations (paracentric inversion/translocation) of the *anaplastic lymphoma kinase* (*ALK*) gene occur in about 3–4% of non-small cell lung cancers (NSCLC) and represent a drugable target [1, 2]. Since the approval of the first ALK tyrosine kinase inhibitor (TKI) in the US (2011) and in Europe (2012), as well as of further second and third line TKI, ALK testing in locally advanced or metastatic non-pure squamous NSCLC is currently the diagnostic standard [3–5]. Fluorescence in-situ hybridization (FISH) was used as the diagnostic method of choice in the studies leading to the approval of Crizotinib [6, 7]. The Food and Drug Administration (FDA) reflected this aspect within the authorization text [8, 9] therefore FISH is still regarded as the gold standard for the detection of ALK rearrangements. However, meanwhile the FDA further approved the Ventana ALK (D5F3) CDx immunohistochemistry (IHC) assay as a companion diagnostics to identify patients eligible for ALK TKI treatment [10, 11]. Thus, two different diagnostic approaches (DNA and protein-based) might be used, if carefully validated [3], for patient care. This is in line with the European Medicines Agency (EMA) authorization text that “only” requires the proof of an “advanced ALK positive NSCLC” [12]. However, even though validated and recommended [3, 13–18], IHC and FISH might still produce discordant results [18–23] leading to a diagnostic and therapeutic dilemma. As RNA-based assays were reported as promising tools for ALK testing in some case series [24–27], we performed massive parallel sequencing (MPS) using IonTorrent chemistry and a probe-based technology (NanoString) allowing a direct counting of RNA molecules in an IHC and FISH pretested NSCLC cohort [13, 21].

## Methods

### ALK NSCLC samples

In this study (ML39478) we refer to already ALK tested (IHC and FISH) NSCLC samples being part of recent publications [13, 21] as well as of our daily routine setting. Therefore, the number of samples included in this study was related to the availability of ALK positive samples. The methods of ALK IHC (VENTANA ALK (D5F3) CDx Assay, Ventana Medical Systems, Tucson, AZ, USA) and ALK FISH (Vysis LSI ALK Dual Color, Abbott Molecular, Abbott Park, IL, USA) were described elsewhere in detail [13, 21, 28–30]. Besides clear cut ALK negative and positive samples by IHC and FISH, a special focus was set on ALK FISH-borderline (BL) samples with split signal (SS)/single red signal (SRS) between 10 and 20%, which were further sub-classified in FISH-BL-negative (10–14% SS/SRS) and FISH-BL-positive (15–20%) [21]. Furthermore, three IHC-negative/FISH-positive tumors (one case with SS/SRS in 30% of the tumor cell nuclei and two cases with > 60% SRS), as well as one with an unclear ALK IHC staining pattern were selected. In total, representative tumor areas of the 33 formalin-fixed and paraffin embedded (FFPE) NSCLC samples (18 operative and 15 biopsy specimen; 32 adenocarcinoma, 1 adeno-squamous carcinoma) were chosen for further RNA-based testing. The different diagnostic ALK-groups were as follows: 12x IHC-negative/FISH-negative, 4x IHC-negative/FISH-BL-negative, 5x IHC-negative/FISH-BL-positive, 3x IHC-negative/FISH-positive, 2x IHC-positive/FISH-BL-positive, 6x IHC-positive/FISH-positive and 1x IHC-unclear/FISH-BL-positive (Table 1).

**Table 1:** All depicted samples were analyzed on transcriptional level with massive parallel sequencing (IonTorrent); *samples additionally analyzed with digital probe-based technology (NanoString)

IHC-negative/FISH-negative	IHC-negative/FISH-BL-negative (10–14%)	IHC-negative/FISH-BL-positive (15–20%)	IHC-negative/FISH-positive (> 30%)	IHC-positive/FISH-BL-positive (15–20%)	IHC-positive/FISH-positive
P01	P27*	P22*	P31*	P19* (IHC uncertain)	P13*
P02*	P28*	P23*	P32*	P20*	P14*
P03*	P29*	P24*	P33*	P21*	P15*
P04*	P30*	P25*			P16*
P05*		P26*			P17
P06					P18
P07					
P08					
P09					
P10					
P11					
P12					
*N* = 12	*N* = 4	*N* = 5	*N* = 3	N = 3	*N* = 6
33 samples					

### RNA-based ALK fusion detection

For ALK fusion detection at the RNA level all 33 samples were subjected to PCR-based MPS. 23 samples were further examined using NanoString allowing a direct count of RNA transcripts.

#### RNA extraction

Three to ten 10 μm thick sections of FFPE tissue were macrodissected and subjected to RNA isolation using the Maxwell16 LEV RNA FFPE Kit (Promega, Fitchburg, WI, USA) according to manufacturers’ protocol modified as follows: samples were incubated with 25 μl proteinase K (20 mg/ml, Promega) and 250 μl lysis buffer (Promega) for 30 min at 56 °C, followed by 25 min of inactivation at 80 °C. No mineral oil was used for RNA extraction procedure.

The RNA concentration was determined using Qubit 2.0 fluorometer (Thermo Fisher Scientific, Waltham, MA, USA) and RNA quality was assessed using the Fragment Analyzer (Advanced Analytical Technologies, Ankeny, IA, USA) microfluidic system.

To determine the limit of detection (LoD) of MPS and the probe-based assay, we serially diluted RNA from the cancer cell line H2228 positive for ALK fusion (EML4-ALK v3a/b) with decreasing amounts of wild-type RNA from palatine tonsil tissue (100, 50, 30, 10, 5, 1 and 0%).

#### Fusion detection with massive parallel sequencing

In order to selectively amplify ALK fusion transcripts, the Ion AmpliSeq RNA Fusion Lung Cancer Research Panel (Thermo Fisher Scientific) was used. Sample enrichment and library preparation followed the instructions of the Ion AmpliSeq DNA and RNA Library Preparation manual (MAN0006735, Revision B.0; Thermo Fisher Scientific). Briefly, a total of 10 ng RNA was reverse transcribed and subjected to target enrichment by multiplex-PCR amplification with Ion AmpliSeq Library Kit 2.0 (Thermo Fisher Scientific). Finally, libraries were quantified by quantitative real-time PCR (Ion Library TaqMan Quantitation Kit, Thermo Fisher Scientific) and pooled in an equimolar ratio. For sequencing, libraries were pooled and templated using the Ion Chef instrument according to the Ion PGM Sequencing 200 Kit v2 user guide or Ion 520 & 530 Kit – Chef user guide (Thermo Fisher Scientific). Subsequently, sequencing of the Ion 318 chip was carried out on the Personal Genome Machine (PGM, Thermo Fisher Scientific) using the Ion Torrent 200 bp sequencing v2 chemistry. Sequencing of the Ion 530 chip was done on the Ion S5 XL system (Thermo Fisher Scientific) following manufacturers’ protocol.

Raw sequencing data was processed using the Torrent Suite Software (version 5.0.2) and aligned against the human genome (version hg19). Detection of fusion transcripts was done using the AmpliSeq RNA Lung Fusion Single Sample High Sensitivity workflow (version 5.0) integrated in the Ion Reporter 5.0.

Data analysis was further performed according to parameters defined by the OncoNetwork Consortium and as described in the guidelines to interpret 3′/5′ imbalance values in the Ion Reporter Software.

#### Fusion detection using digital probe-based analysis

nCounter Lung Gene Fusion Panel (NanoString Technologies, Seattle, WA, USA) was used for probe-based detection of fusion genes in the selected tumor samples. Hybridization and digital reporter counts were performed following the manufacturer’s instructions for nCounter Vantage Fusion Assays (MAN-10023-09, nCounter XT Assay User Manual). Briefly, 84 ng–300 ng of total FFPE RNA was hybridized to nCounter probe sets for 16 h at 67 °C. Samples were processed using automated nCounter Sample Prep Station (NanoString Technologies). Cartridges were subsequently imaged on an nCounter Digital Analyzer (NanoString Technologies) set at 555 fields of view (FoV). Reporter counts were collected using NanoString’s NSolver software version 3.0 and analyzed with fusion threshold set at 50 and imbalance threshold at 15 depending on the background level of absolute raw data count.

## Results

### ALK rearrangement detection by sequencing

#### Defining limit of detection with cell line dilution series

MPS fusion breakpoint detection showed a LoD at 10% fusion positive cell line dilution, whereas EML4-ALK fusion detection by 3′/5′ imbalance was uncertain or negative for all H2228 dilutions (Table 2). The MPS assay showed 100% specificity for ALK fusion detection and a higher sensitivity for breakpoint than for 3′/5′ analysis (67% vs. 33%) with uncertain data stated as negative.

**Table 2:** Limit of detection, sensitivity, specificity and accuracy of ALK fusion detection with massive parallel sequencing (IonTorrent) and a probe-based assay (NanoString) determined using a cell line dilution series

	Massive Parallel Sequencing (MPS)		Probe-Based Assay	
	ALK 3′/5’ Imbalance	ALK Fusion	ALK 3′/5’ Imbalance	ALK Fusion
H2228 Cell Line Dilution in Palatine Tonsil Background				
100%	Uncertain	Yes	Yes	Yes
50%	Uncertain	Yes	Yes	Yes
30%	Uncertain	Yes	Yes	Yes
10%	Uncertain	Yes	Yes	No
5%	No	No	Yes	No
1%	No	No	No	No
0%	No	No	No	No
Assay Parameter				
Sensitivity	33.3%	66.7%	83.3%	50.0%
Specificity	100%	100%	100%	100%
NPV	20.0%	33.3%	50.0%	25.0%
PPV	100%	100%	100%	100%
Accuracy	42.9%	71.4%	85.7%	57.1%

*NPV* negative predictive value, *PPV* positive predictive value

#### Analyzing clinical samples

Imbalance and fusion breakpoint results were concordant in 29/32 analyzable cases (90%). Variations were detected for imbalance results in which 4/32 cases were uncertain, two were fusion breakpoint negative after repetition and one remained uncertain. Imbalance result for 1/32 sample, detected as fusion breakpoint positive was negative after repetition.

### Identification of ALK fusions by digital probe-based assay

#### Defining limit of detection with cell line dilution series

ALK fusion was detected by 3′/5′ imbalance probes down to 5% cell line dilution whereas LoD for fusion detection by breakpoint probe was 30% (Table 2).

The probe-based assay showed 100% specificity for ALK fusion detection and a higher sensitivity for 3′/5′ analysis than for breakpoint detection (83% vs. 50%).

#### Analyzing clinical samples

Imbalance and fusion breakpoint results of clinical samples were concordant in all 23 cases (100%).

### RNA based analysis of unequivocal and equivocal (discordant) ALK cases

Eighteen unequivocal and 15 equivocal samples were investigated by MPS and/or a digital probe-based method. Four out of 12 ALK IHC-negative/FISH-negative and all 6 IHC-positive/FISH-positive cases showed concordance with both RNA-based methods regarding fusion breakpoint detection, one case was not analyzable (Table 3).

**Table 3:** RNA-based analysis of unequivocal ALK cases showed 100% concordance between digital probe-based assay and IHC and FISH results. ALK fusion detection based on 3′/5′ imbalance MPS assay showed 25% deviation for IHC-negative/FISH-negative cases and 33% deviation for IHC-positive/FISH-positive cases. Data showed 92% concordance between ALK fusion breakpoint detection by MPS assay and IHC and FISH for IHC-negative/FISH-negative samples and 100% accordance for IHC-positive/FISH-positive samples

Sample	IHC	FISH	Massive Parallel Sequencing (MPS) (IonTorrent)				Probe-Based Assay (NanoString)		Fusion
			Fusion		3′/5’		Fusion	3′/5’	
IHC-negative/FISH-negative									
P01	**–**	**–**	NA	NA	NA	NA	ND		
P02	**–**	**–**	**–**	**–**	±	**–**	**–**	**–**	
P03	**–**	**–**	**–**	**–**	±	**–**	**–**	**–**	
P04	**–**	**–**	**–**		**–**		**–**	**–**	
P05	**–**	**–**	**–**		**–**		**–**	**–**	
P06	**–**	**–**	**–**		**–**		ND		
P07	**–**	**–**	**–**		**–**		ND		
P08	**–**	**–**	**–**		**–**		ND		
P09	**–**	**–**	**–**		**–**		ND		
P10	**–**	**–**	**–**		**–**		ND		
P11	**–**	**–**	**–**		**–**		ND		
P12	**–**	**–**	**–**		**–**		ND		
IHC-positive/FISH-positive									
P13	**+**	**+**	**+**	**+**	**+**	**–**	**+**	**+**	V1
P14	**+**	**+**	**+**		**±**		**+**	**+**	V1
P15	**+**	**+**	**+**		**+**		**+**	**+**	V3a/b
P16	**+**	**+**	**+**		**+**		**+**	**+**	V2
P17	**+**	**+**	**+**		**+**		ND		V1
P18	**+**	**+**	**+**	**+**	**+**	**+**	ND		V6

Fusion: fusion detection with amplicons/probes covering ALK fusion breakpoint; 3′/5′: fusion detection with amplicons/probes covering 3′ and 5′ end; NA: Not Analyzable; ND: Not Determined; +: fusion detected; −: fusion not detected; ±: fusion uncertain; V1: EML4_13:ALK_20; V2: EML4_20:ALK_20; V3a: EML4_6a:ALK_20; V3b: EML4_6b:ALK_20; V6: EML4_13:ALK_20_ins69

The three IHC-negative/FISH-positive samples were negative with the two RNA-based methods for both, breakpoint and imbalance detection (Table 4).

**Table 4:** RNA-based analysis of equivocal ALK cases; 75% (6/8) of FISH-BL-positive cases were negative, 25% (2/8) were positive with both RNA-based methods. All FISH-BL-negative cases were ALK fusion negative with MPS and digital probe-based assay

Sample	IHC	FISH	Massive Parallel Sequencing (MPS) (IonTorrent)				Probe-Based Assays (NanoString)		Fusion
			Fusion		3′/5′		Fusion	3′/5′	
IHC-uncertain/FISH-BL-positive									
P19	**±**	**+**	**–**		**–**		**–**	**–**	
IHC-positive/FISH-BL-positive									
P20	**+**	**+**	**+**	**+**	**±**	**±**	**+**	**+**	V3a/b
P21	**+**	**+**	**+**		**+**		**+**	**+**	V1
IHC-negative/FISH-BL-positive									
P22	**–**	**+**	**–**		**–**		**–**	**–**	
P23	**–**	**+**	**–**	NA	**–**	NA	**–**	**–**	
P24	**–**	**+**	**–**		**–**		**–**	**±**	
P25	**–**	**+**	**–**		**–**		**–**	**–**	
P26	**–**	**+**	**–**		**–**		**–**	**–**	
IHC-negative/FISH-BL-negative									
P27	**–**	**–**	**–**	**–**	**–**	**–**	**–**	**–**	
P28	**–**	**–**	**–**		**–**		**–**	**–**	
P29	**–**	**–**	**–**		**–**		**–**	**–**	
P30	**–**	**–**	**–**		**–**		**–**	**–**	
IHC-negative/FISH-positive									
P31	**–**	**+**	**–**		**–**		**–**	**–**	
P32	**–**	**+**	**–**		**–**		**–**	**–**	
P33	**–**	**+**	**–**		**–**		**–**	**–**	

**Fusion:** fusion detection with amplicons/probes covering ALK fusion breakpoint; **3′/5′:** fusion detection with amplicons/probes covering 3′ and 5′ end; **NA:** Not Analyzable; **ND:** Not Determined; **+:** fusion detected; **−:** fusion not detected; **±:** fusion uncertain; **V1:** EML4_13:ALK_20; **V3a:** EML4_6a:ALK_20; **V3b:** EML4_6b:ALK_20

**Fig. 1:**
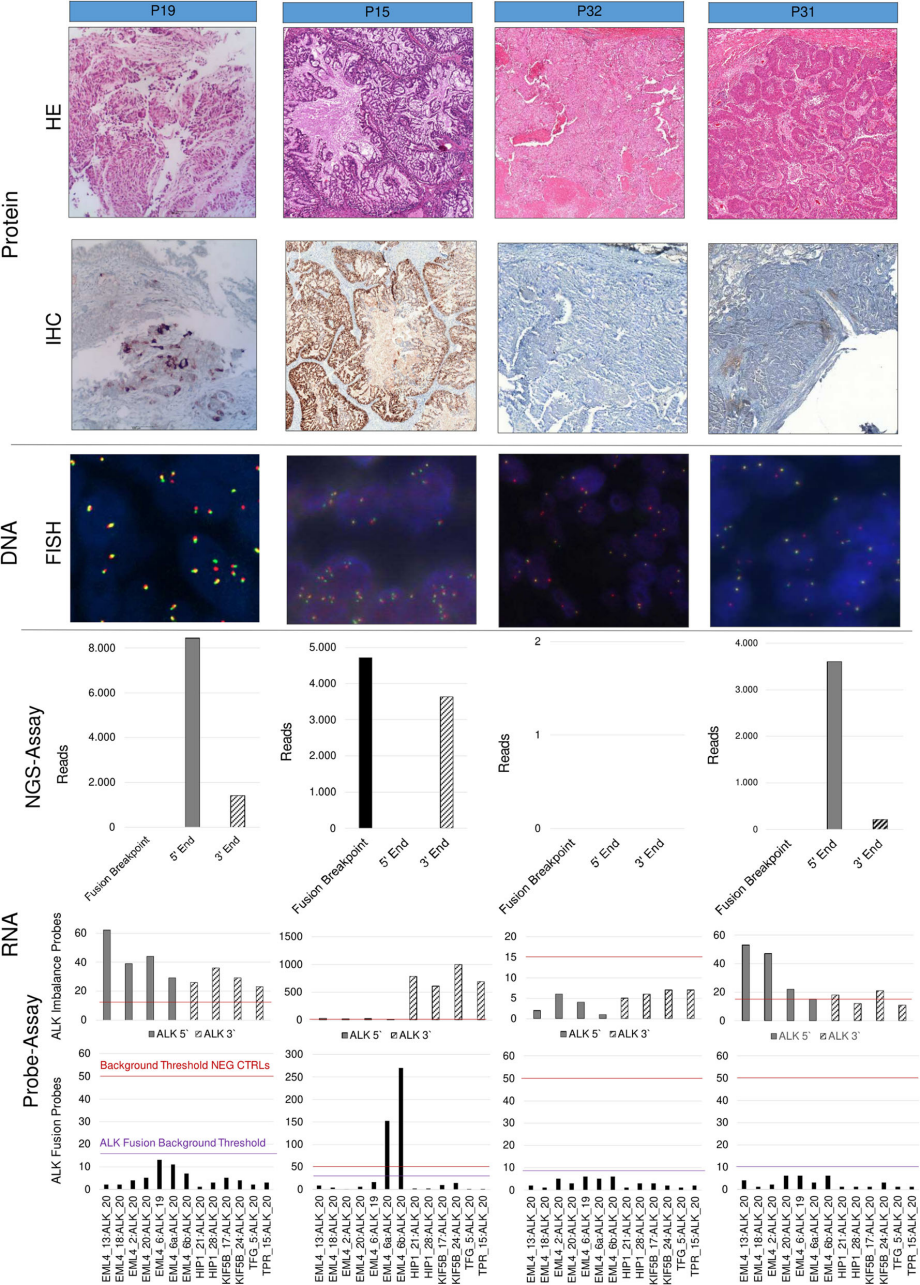
Samples tested for ALK fusions on protein, DNA and RNA level. Sample P19 shows ALK IHC uncertain and FISH-BL-positive results, whereas results on transcriptional level were negative. P15 is concordantly positive with all applied methods. P32 and P31 are both ALK IHC-negative/ FISH-positive. These samples are ALK fusion negative on transcriptional level with both RNA-based methods (NanoString and IonTorrent), whereas P32 shows no RNA count, indicating no ALK expression. HE: hematoxylin and eosin staining; IHC: immunohistochemistry; FISH: fluorescence in-situ hybridization: MPS: Massive Parallel Sequencing; NEG CTRLs: negative controls; Probe-assay data shown are absolute raw data counts

The four IHC-negative/FISH-BL-negative cases, as well as the five IHC-negative/FISH-BL-positive samples showed negative results by MPS and digital probe-based assay. The two IHC-positive/FISH-BL-positive samples were consistently positive at the RNA level, whereas the case showing questionable IHC and FISH-BL-positive status was negative (Fig. 1).

## Discussion

Since the approval of Crizotinib, there had been much effort to improve, harmonize and even fasten the diagnostic process of ALK testing [13–16, 31–33]. In this context, several ring trials and multi-center studies have confirmed the diagnostic reliability and quality of IHC and FISH [13–16, 31–34]. Concerning in-situ hybridization it was further shown that CISH (chromogenic based) can be regarded as an alternative reliable method [35, 36]. However, due to technical reasons, there might be samples that will be misclassified if FISH or CISH was used as stand-alone test [21, 37]. This is especially true for samples that show SS/SRS around the 15% cutoff [21]. To this end, it was highly appreciated that several initiatives demonstrated ALK IHC as a dependable method, if carefully validated [10, 13, 15, 31–33, 38–40]. However, there are still several reports depicting discrepant results by IHC and FISH [18–26], leading to diagnostic problem and a potential “wrong” therapeutically decision. First of all, this is due to the so-called BL tumors especially those with SS/SRS FISH signals between 15 and 20% of the tumor cells. The fact that “true” ALK negative tumors and normal lung tissue may harbor SS in up to 11% of the tumor cells and ~ 8.5% of the NSCLC show SS between 10 and 15% is further complicating this situation [41–43]. To this end it was recommended, that in biopsy specimens (with a few tumor cells only), the unequivocal result of a validated IHC should be used for therapy planning in NSCLC cases even though if FISH was negative [22].

Our data underline that a validated ALK IHC produces dependable results in most cases. We only found one sample in our cohort leading to an unclear staining pattern due to some, focal positive cells (questionable tumor cells or macrophages). The Ventana Interpretation Guide for the D5F3-Optiview system defines IHC-positivity if even single tumor cells show a cytoplasmic staining [28]. However, in our experience ALK positive staining, if truly positive, will be found in all (almost all) tumor cells and not merely in a small subpopulation due to its role as driver mutation. This is further demonstrated by the homogenous staining for the ALK protein in all tumor cells of the IHC-positive/FISH-BL-positive cases and further underlines that the occurrence of discrepant ALK results for IHC and FISH is due to technical reasons of FISH. This assumption was confirmed by some studies, which were able to reproduce the IHC results on the RNA-level by means of RT-PCR or MPS [24–27, 44, 45]. This is also true for our study that confirmed the IHC results by MPS and the probe-based NanoString assay and therefore strengthens the hypothesis that IHC and RNA-based methods reflect the true biological situation. Unfortunately, clinical data within our cohort were scarce for the most of the cases. However, at present four patients of our study with an ALK IHC-negative/FISH-BL status were treated with an ALK TKI at Charité University Hospital and all did not show any benefit based on this therapy.

Interestingly, we further characterized three other discrepant samples, which were ALK IHC-negative/FISH-positive and also negative at the transcriptional level by MPS and NanoString. Concerning the ALK FISH pattern, two of them displayed SRS in > 60% of the tumor cells. A comparable observation, with SRS-samples displaying no protein expression and no transcript was made by Gao et al. [45]. Therefore, it seems that IHC/FISH discrepancy might be caused in the context of a FISH BL-status, especially in the context of small biopsies [22], however, might be further seen in at first sight “true” FISH-positive cases with SRS-pattern. This situation is still insufficiently covered by clinical data but it is justified to conclude that no transcription or translation of the ALK fusion gene occurs [45, 46]. A possible explanation for this observation, the break might have occurred but the promoter might not have been translocated to the *ALK* gene or might even have been lost, thus preventing *ALK* expression despite the genomic *ALK* alteration (non-productive rearrangement). This might be one explanation for the documented non-responders in the FISH-based trials leading to the TKI approval (see waterfall blot in ref. [6, 7]) already discussed in the literature [47].

To conclude, as predictive testing in NSCLC becomes more and more complex and further treatable targets (besides EGFR, ALK, ROS1, MET, RET, BRAF, HER2, PD-L1) will arise in the nearer future [2], pragmatic approaches (reliable, not time and money consuming, multiplexable) are needed. Furthermore, current hybrid capture-based sequencing assays allow the additional detection of so far unknown fusion partners [48–50]. As this might not always be applicable for routine analysis, this was beyond the scope of our investigations. However, these approaches will enable a further comprehensive fusion analysis, helping for a better understanding of the molecular mechanism in lung cancer [48–51]. The results of this study show that ALK testing should be based on methods that reflect the functional level of ALK. As RNA-based methods confirmed the IHC-status, future diagnostic algorithms should be based on these approaches, whereas FISH, at least as a stand-alone test, seems not eligible anymore.

## Conclusions

The comparison of different methods for the confirmation of ALK rearrangements revealed that the detection of the protein (IHC) and the fusion transcripts on transcriptional level (sequencing and probe-based assays) leads to concordant results. A small proportion of clearly ALK FISH-positive cases do not express the ALK protein and the fusion transcript, which might explain non-response to ALK inhibitors (non-productive rearrangement). Therefore, our findings led us to conclude that ALK testing should initially be based on IHC- and/or RNA-based methods. This might further avoid needless ALK-TKI therapies in ALK FISH-BL cases.

## Abbreviations

ALK: Anaplastic lymphoma kinase
BL: Borderline
FISH: Fluorescence in-situ hybridization
IHC: Immunohistochemistry
LoD: Limit of detection
MPS: Massive parallel sequencing
NSCLC: Non-small cell lung cancer
SRS: Single red signal
SS: Split signal

## References

[1] Soda M, Choi YL, Enomoto M, Takada S, Yamashita Y (2007). Identification of the transforming EML4-ALK fusion gene in non-small-cell lung cancer. Nature.

[2] Thunnissen E, Allen TC, Adam J, Aisner DL, Beasley MB (2018). Immunohistochemistry of pulmonary biomarkers: a perspective from members of the pulmonary pathology society. Arch Pathol Lab Med.

[3] Lindeman NI, Cagle PT, Beasley MB, Chitale DA, Dacic S (2013). Molecular Testing Guideline for Selection of Lung Cancer Patients for EGFR and ALK Tyrosine Kinase Inhibitors: Guideline from the College of American Pathologists, International Association for the Study of Lung Cancer, and Association for Molecular Pathology. Arch Pathol Lab Med.

[4] Kerr KM, Bubendorf L, Edelman MJ, Marchetti A, Mok T (2014). Second ESMO consensus conference on lung cancer: pathology and molecular biomarkers for non-small-cell lung cancer. Ann Oncol.

[5] Thunnissen E, Bubendorf L, Dietel M, Elmberger G, Kerr K (2012). Consensus statement on testing for EML4-ALK in non-small-cell carcinomas of the lung. Virch Arch.

[6] Shaw AT, Yeap BY, Solomon BJ, Riely GJ, Gainor J (2011). Effect of crizotinib on overall survival in patients with advanced non-small-cell lung cancer harbouring ALK gene rearrangement: a retrospective analysis. Lancet Oncol.

[7] Kwak EL, Bang YJ, Camidge DR, Shaw AT, Solomon B (2010). Anaplastic lymphoma kinase inhibition in non-small-cell lung cancer. N Engl J Med.

[8] https://www.cancer.gov/about-cancer/treatment/drugs/fda-crizotinib Accessed 19 Feb 2018.

[9] https://www.accessdata.fda.gov/cdrh_docs/pdf14/p140025b.pdf Accessed 19 Feb 2018.

[10] Marchetti A, Di Lorito A, Pace MV, Iezzi M, Felicioni L (2016). ALK Protein Analysis by IHC Staining after Recent Regulatory Changes: A Comparison of Two Widely Used Approaches, Revision of the Literature, and a New Testing Algorithm. J Thorac Oncol.

[11] https://www.accessdata.fda.gov/cdrh_docs/pdf16/p160045d.pdf Accessed 19 Feb 2018.

[12] http://www.ema.europa.eu/docs/en_GB/document_library/EPAR_-_Public_assessment_report/human/002489/WC500134761.pdf Accessed 19 Feb 2018.

[13] von Laffert M, Warth A, Penzel R, Schirmacher P, Kerr KM (2014). Multicenter Immunohistochemical ALK-Testing of Non-Small-Cell Lung Cancer Shows High Concordance after Harmonization of Techniques and Interpretation Criteria. J Thorac Oncol.

[14] Nitta H, Tsuta K, Yoshida A, Ho SN, Kelly BD (2013). New methods for ALK status diagnosis in non-small-cell lung cancer: an improved ALK immunohistochemical assay and a new, Brightfield, dual ALK IHC-in situ hybridization assay. J Thorac Oncol.

[15] Wynes MW, Sholl LM, Dietel M, Schuuring E, Tsao MS (2014). An international interpretation study using the ALK IHC antibody D5F3 and a sensitive detection kit demonstrates high concordance between ALK IHC and ALK FISH and between evaluators. J Thorac Oncol.

[16] Savic S, Diebold J, Zimmermann AK, Jochum W, Baschiera B (2015). Screening for ALK in non-small cell lung carcinomas: 5A4 and D5F3 antibodies perform equally well, but combined use with FISH is recommended. Lung Cancer.

[17] Sholl LM, Weremowicz S, Gray SW, Wong KK, Chirieac LR (2013). Combined Use of ALK Immunohistochemistry and FISH for Optimal Detection of ALK-Rearranged Lung Adenocarcinomas. J Thorac Oncol.

[18] von Laffert M, Schirmacher P, Warth A, Weichert W, Büttner R (2017). ALK-Testing in non-small cell lung cancer (NSCLC): Immunohistochemistry (IHC) and/or fluorescence in-situ Hybridisation (FISH)?: Statement of the German Society for Pathology (DGP) and the Working Group Thoracic Oncology (AIO) of the German Cancer Society e.V. (Stellungnahme der Deutschen Gesellschaft für Pathologie und der AG Thorakale Onkologie der Arbeitsgemeinschaft Onkologie/Deutsche Krebsgesellschaft e.V.). Lung Cancer.

[19] Ilie MI, Bence C, Hofman V, Long-Mira E, Butori C (2015). Discrepancies between FISH and immunohistochemistry for assessment of the ALK status are associated with ALK ‘borderline’-positive rearrangements or a high copy number: a potential major issue for anti-ALK therapeutic strategies. Ann Oncol.

[20] Ilie M, Hofman P (2015). Reply to the letter to the editor “ALK FISH rearranged and amplified tumor with negative immunohistochemistry: a rare and challenging case concerning ALK status screening in lung cancer” by Uguen et al. Ann Oncol.

[21] von Laffert M, Stenzinger A, Hummel M, Weichert W, Lenze D (2015). ALK-FISH borderline cases in non-small cell lung Cancer: implications for diagnostics and clinical decision making. Lung Cancer.

[22] Abe H, Kawahara A, Azuma K, Taira T, Takase Y (2015). Heterogeneity of anaplastic lymphoma kinase gene rearrangement in non-small-cell lung carcinomas: a comparative study between small biopsy and excision samples. J Thorac Oncol.

[23] Minca EC, Portier BP, Wang Z, Lanigan C, Farver CF (2013). ALK status testing in non-small cell lung carcinoma: correlation between ultrasensitive IHC and FISH. J Mol Diagn.

[24] Peled N, Palmer G, Hirsch FR, Wynes MW, Ilouze M (2012). Next-generation sequencing identifies and immunohistochemistry confirms a novel Crizotinib-sensitive ALK Rearrangemnet in a patient with metastatic non-small-cell lung Cancer. J Thorac Oncol.

[25] Ren S, Hirsch FR, Varella-Garcia M, Aisner DL, Boyle T (2014). Atypical negative ALK break-apart FISH harboring a crizotinib-responsive ALK rearrangement in non-small-cell lung cancer. J Thorac Oncol.

[26] Pekar-Zlotin M, Hirsch FR, Soussan-Gutman L, Ilouze M, Dvir A (2015). Fluorescence in situ hybridization, immunohistochemistry, and next-generation sequencing for detection of EML4-ALK rearrangement in lung cancer. Oncologist.

[27] Pfarr N, Stenzinger A, Penzel R, Warth A, Dienemann H (2016). High-throughput diagnostic profiling of clinically actionable gene fusions in lung cancer. Genes Chromosomes Cancer.

[28] http://www.roche-diagnostics.ch/content/dam/corporate/roche-dia_ch/documents/broschueren/tissue_diagnostics/Parameter/lung-pathology/ALK_D5F3_interpretation%20Guide_EN.pdf Accessed 20 Aug 2017.

[29] https://www.molecular.abbott/sal/en-us/staticAssets/Vysis_ALK_Evaluation_Guide_Final.pdf Accessed 20 Aug 2017.

[30] Yi ES, Boland JM, Maleszewski JJ, Roden AC, Oliveira AM (2011). Correlation of IHC and FISH for ALK gene rearrangement in non-small cell lung carcinoma: IHC score algorithm for FISH. J Thorac Oncol.

[31] Blackhall FH, Peters S, Bubendorf L, Dafni U, Kerr KM (2014). Prevalence andclinical outcomes for patients with ALK-positive resected stage I to III adenocarcinoma: results from the European thoracic oncology platform Lungscape project. J Clin Oncol Off J Am Soc Clin Oncol.

[32] Cutz JC, Craddock KJ, Torlakovic E, Brandao G, Carter RF (2014). Canadian anaplastic lymphoma kinase study: a model for multicenter standardization and optimization of ALK testing in lung cancer. J Thorac Oncol.

[33] Selinger CI, Rogers TM, Russell PA, O'Toole S, Yip P (2013). Testing for ALK rearrangement in lung adenocarcinoma: a multicenter comparison of immunohistochemistry and fluorescent in situ hybridization. Mod Pathol.

[34] von Laffert M, Penzel R, Schirmacher P, Warth A, Lenze D (2014). Multicenter ALK Testing in Non-Small-Cell Lung Cancer: Results of a Round Robin Test. J Thorac Oncol.

[35] Wagner F, Streubel A, Roth A, Stephan-Falkenau S, Mairinger T (2014). Chromogenic in situ hybridisation (CISH) is a powerful method to detect ALK-positive non-small cell lung carcinomas. J Clin Pathol.

[36] Schildhaus HU, Deml KF, Schmitz K, Meiboom M, Binot E (2013). Chromogenic in situ hybridization is a reliable assay for detection of ALK rearrangements in adenocarcinomas of the lung. Mod Pathol.

[37] McLeer-Florin A, Lantuéjoul S (2012). Why technical aspects rather than biology explain cellular heterogeneity in ALK-positive non-small cell lung cancer. J Thorac Dis.

[38] Hutarew G, Hauser-Kronberger C, Strasser F, Llenos IC, Dietze O (2014). Immunohistochemistry as a screening tool for ALK rearrangement in NSCLC: evaluation of five different ALK antibody clones and ALK FISH. Histopathology.

[39] Conde E, Suárez-Gauthier A, Benito A, Garrido P, García-Campelo R (2014). Accurate identification of ALK positive lung carcinoma patients: novel FDA-cleared automated fluorescence in situ hybridization scanning system and ultrasensitive immunohistochemistry. PLoS One.

[40] Alì G, Proietti A, Pelliccioni S, Niccoli C, Lupi C (2014). ALK rearrangement in a large series of consecutive non-small cell lung cancers: comparison between a new immunohistochemical approach and fluorescence in situ hybridization for the screening of patients eligible for crizotinib treatment. Arch Pathol Lab Med.

[41] Camidge DR, Kono SA, Flacco A, Tan AC, Doebele RC (2010). Optimizing the detection of lung cancer patients harboring anaplastic lymphoma kinase (ALK) gene rearrangements potentially suitable for ALK inhibitor treatment. Clin Cancer Res.

[42] McLeer-Florin A, Moro-Sibilot D, Melis A, Salameire D, Lefebvre C (2012). Dual IHC and FISH testing for ALK gene rearrangement in lung adenocarcinomas in a routine practice: a French study. J Thorac Oncol.

[43] Camidge DR, Skokan M, Kiatsimkul P, Helfrich B, Lu X (2013). Native and rearranged ALK copynumber and rearranged cell count in non-small cell lung cancer: implicationsfor ALK inhibitor therapy. Cancer.

[44] Jung Y, Kim P, Jung Y, Keum J, Kim SN (2012). Discovery of ALK-PTPN3 gene fusion from human non-small cell lung carcinoma cell line using next generation RNA sequencing. Genes Chromosomes Cancer.

[45] Gao X, Sholl LM, Nishino M, Heng JC, Jänne PA (2015). Clinical Implications of Variant ALK FISH Rearrangement Patterns. J Thorac Oncol.

[46] Volckmar AL, Endris V, Bozorgmehr F, Lier C, Porcel C (2016). Next-generation sequencing facilitates detection of the classic E13-A20 EML4-ALK fusion in an ALK-FISH/IHC inconclusive biopsy of a stage IV lung cancer patient: a case report. Diagn Pathol.

[47] Chihara D, Suzuki R (2011). More on crizotinib. N Engl J Med.

[48] Heuckmann JM, Pauwels P, Thunnissen E (2017). Comprehensive hybrid capture-based next-generation sequencing identifies a double ALK gene fusion in a patient previously identified to be false-negative by FISH. J Thorac Oncol.

[49] Plenker D, Bertrand M, de Langen AJ, Riedel R, Lorenz C (2018). Structural alterations of MET kinase inhibition in lung adenocarcinoma patients. Clin Cancer Res.

[50] Rosoux A, Pauwels P, Duplaquet F, D’Haene N, Weynand B (2016). Effectiveness of crizotinib in a patient with ALK IHC-positive/FISH-negative metastatic lung adenocarcinoma. Lung Cancer.

[51] Vendrell JA, Taviaux S, Béganton B, Godreuil S, Audran P (2017). Detection of known and novel ALK fusion transcripts in lung cancer patients using next-generation sequencing approaches. Sci Rep.

